# Ethnicity and risk factors among Indian coronary artery disease patients

**DOI:** 10.6026/97320630019019

**Published:** 2023-01-31

**Authors:** Monika Sah, Latheef SAA, Venkataramana P

**Affiliations:** 1 Discipline of Anthropology, School of Social Sciences, Indira Gandhi National Open University, Maidan Garhi, New Delhi-110068, India; 2 Department of Genetics, Osmania University, Hyderabad, India

**Keywords:** Coronary artery disease, heart disease risk factors, social class

## Abstract

In this study, an attempt was made to investigate the distribution of coronary risk factors in male patients with coronary artery disease (CAD)(n=50 each) belonging to Jaat and Vaishya castes. A Significantly higher average height, waist and hip circumferences, glucose, and waist-height ratio were observed in Jaats compared to the Vaishyas (p=0.000). Mean BMI, total cholesterol (total-C) and non-high density lipoprotein cholesterol (HDL-C), and lean body mass index (LBMI) were significantly higher in Vaishyas against Jaats (p=0.00). A significantly higher percentage of type 2 diabetes(T2DM) (p=0.03) and isolated hypertriglyceridemia(p=0.01) was observed in Jaats against Vaishya men. Percentage of general obesity(p=0.01), high total-C, high low density lipoprotein cholesterol (LDL-C) (p=0.00), high total-C/HDL-C(p=0.04), combined positive family history of hypertension and type 2 diabetes, and general obesity, was significantly higher in Vaishya when compared to Jaat men. In univariate logistic regression analyses, a significant association of T2DM (p=0.039) and isolated hypertriglyceridemia (p=0.020) with Jaat ethnic group and general obesity, high totalC, high LDL-C, and high total-C/HDL-C with Vaishya ethnic group was observed. Results of the present study suggest that a population-specific than a global approach should be used in identifying high-risk groups and designing of interventions to reduce the complications and management of CAD.

## Background:

Analysis of the global burden disease dataset from 1990-2016 revealed that 126 million or 1.72% world's population were affected by CAD and responsible for nine million global deaths [1]. Higher age standardized CVD deaths rate in India than the global average death rate (272/100000 vs 235/100000 population) and a wide variation in the prevalence of coronary risk factors were reported [2]. The prevalence of CAD increased from 2% to 14% in urban areas and from 1.7% to 7.4% in rural areas in India [3]. Risk factors such as smoking, diabetes, hypertension, abdominal obesity, psychosocial stress, physical inactivity, dyslipidaemia, and unhealthy diet were identified as CAD risk factors in India's case-control studies [4]. Variation in the distribution of coronary risk factors among the ethnic groups was reported in Europe and Iran [5]. Indian population is heterogeneous consisting of 4635 anthropologically defined groups [6]. It may be possible that due to the differential access or restriction to the resources, culture, nutrition, habits and traditions, the burden of coronary risk factors may be different in social groups of India. Investigation of risk factors may be helpful in identifying high-risk groups and initiating tailored interventions in the specific groups [7]. Traditional coronary risk factors such as obesity, insulin resistance, and dyslipidaemia failed to explain heterogeneity in the prevalence of CAD and there is a need to explore the role of ethnicity in the pathophysiology of coronary risk factors [7]. A few studies were conducted on coronary risk factors among healthy caste populations [8,9-10] but not in CAD patients. In this study, an attempt was made to investigate the risk factors of CAD patients among the Jaat and Vaishya caste populations.

## Methodology:

## Sample size:

The estimated sample size required for the study was 50 for two caste populations each when the following assumptions were made: (1) α= 0.05, (2) β= 0.20, (3) mean difference= 8, (4) standard deviation = 14 in two endogamous groups and (5) the number of cases in two endogamous groups are equal.

## Inclusion criteria:

Newly diagnosed CAD patients aged >20 years.

## Exclusion criteria:

Those who were in critical condition and unwilling were excluded.

Patients of CAD (n=50) each belonging to Jaat and Vaishya were recruited in the study following inclusion and exclusion criteria when patients were transferred to intermediate care units of departments of Cardiology of Asian Institute of Medical Sciences and the Fortis Escort hospital Faridabad, Haryana.

CAD was diagnosed based on the angiogram, elevated serum creatine kinase (MB) and electrocardiogram by the cardiologists of the respective hospitals. Data on age, marital status, type of family, education, occupation, income, physical activity, tobacco chewing, alcohol intake, positive family history of CAD and other diseases were enquired from the patients and documented. Socio-economic status of the patients was categorized using the modified KuppuSwamy scale (2021). After inquiring the patients, physical activity was categorized into no, occasional and regular activity. Height was measured on minimal clothing using anthropometer and weight on minimal clothing by calibrated electronic weighing machine. Body mass index (BMI) was calculated using the formula (weight in Kg/ height in meters2). Those patients exceeding BMI values >25 were considered obese. Waist circumference (WC) was measured mid-way between the inferior margin of the last rib and the crest of the ileum in horizontal plane and hip circumference taken at the point of maximal protrusion of buttocks while the patient was standing with his feet close to each other, employing plastic tape. Waist-hip ratio (WHR) was calculated using the formula (waist circumference /hip circumference). Those patients having WHR ratio of 0.9 and WC >90 centimetres were categorized as centrally obese. Blood pressure was recorded using manual Mercury sphygmomanometer. Patients with systolic blood pressure (SBP >140mmHg) and diastolic blood pressure (DBP) >90 mmHg or/and on medication for hypertension were labelled as hypertensive. A 5 mL anticoagulated non-fasting blood sample was drawn from the patients and plasma glucose, total-C, HDL-C, triglyceride and LDL-C levels were estimated using enzymatic kits on Roche autoanalyzer model COBAS integra 400 plus. Those patients with random glucose level of >200mg/dl were labelled as T2DM. CAD patients were classified into different groups using the following criteria: High total-C(>200mg/dl), high triglyceride(>150mg/dl), high LDL-C(>130mg/dl), low HDL-C(<40mg/dl), High LDL-C/HDL-C (>3.5), high total-C/HDL-C(>4.5), isolated hypercholesterolemia(total-C ≥200 mg/dl and triglycerides <150 mg/dl), isolated hypertriglyceridemia(triglycerides ≥150 mg/dl and total-C <200 mg/dl), isolated low HDL cholesterol (men <40; women in the absence of hypertriglyceridemia or hypercholesterolemia) and dyslipidemia (more than one lipid abnormalities).

## Results:

Averages of continuous variables between Jaats and Vaishya were compared to know the significant differences and the results are presented in Table 1. A significantly higher average height, waist and hip circumferences, glucose and waist-height ratio was observed in Jaats compared to the Vaishya. Mean BMI, total and non-HDL cholesterol and lean body mass index were significantly higher in Vaishya against Jaats.The qualitative categorical variables of Jaat and Vaishya men with CAD were compared to find out significant differences and the results are shown in Table 2. Most of the patients in both the ethnic groups were aged >40 years, smokers, alcoholics, married, had joint type of families, graduated, had a monthly income of Rs. 46129-61,662, belonged to upper middle class, were occasionally physically active, consuming gutka and taking non-vegetarian diet. Most of the Jaat patients were involved in business while the majority of Vaishya engaged in private jobs. A significant difference in the patients of both ethnic groups was observed in the type of family (p=0.00) and occupation (p=0.03). A significantly higher percentage of type 2 diabetes and isolated hypertriglyceridemia was observed in Jaats against Vaishya men. Percentage of general obesity, high total-C, high LDL-C, high total-C/HDL-C, combined positive family history of hypertension and type 2 diabetes and general obesity, was significantly higher in Vaishya when compared to Jaat men.

## Discussion:

Studies on the prevalence of coronary risk factors (CRFs) in CAD patients of different ethnic groups [7,8,9, 10,11 and 12], observed differential distribution of CRFs. Prevalence of CRFs in Marwari [13], Bhatia 9] and Agarwal ethnic [14] groups, were reported. No study is available on the distribution of CRFs in CAD patients of ethnic groups in India. The differences in the distribution of CRFs in CAD patients between ethnic groups helps to understand the aetiology of CAD, to identify high-risk groups, and to develop population specific interventions and prevention programmes to reduce the risk of CAD [7]. In the present study, no significant difference in the variables such as income, education and socio-economic status was observed between ethnic groups but other studies found a significant difference [15, 16,]. In contrast to the studies [15-16], our study showed a significant difference in the distribution of occupation levels between the ethnic groups. In agreement with other investigations, our study also showed no significant difference in physical activity levels between ethnic groups [17]. A significant difference in anthropometric and biochemical variables was observed between ethnic groups in the present study as observed in an earlier study [18] except in weight, systolic and diastolic blood pressures, triglyceride and HDL-C. In our study, significant difference in BMI was observed between the ethnic groups like an earlier study [11] but other study failed to show such difference [19]. In Iranian study, mean BMI was higher in Tork (27.62) followed by Mazani (27.52), Fars (27.55), Gilak (27.39), Lor (27.11) and Kord (27.01) [7]. Stepwise discriminant analysis(data not showed) in the present study revealed that variables such as height, BMI, LBM, waist and hip circumferences showed value of more than 0.3 in structure matrix table and these variables were found successfully classify 87% of Jaat and Vaishya patients. To know the effect of qualitative categorical variables on the mean values of anthropometric and biochemical variables, two-way MANOVA was performed. Age and tobacco chewing in both ethnic groups were found to influence the hip circumference (HC) and, waist-height ratio (WHtR) and HC respectively but their contribution were found to vary in both ethnic groups. In addition to HC, tobacco chewing in Jaats was found to influence waist circumference (WC) and WHtR. Influence of occupation on BMI, WC and HC; income on total-C/HDL-C; physical activity on HDL-C and; socio-economic status on glucose was observed. The contribution of qualitative categorical variables on the quantitative variables was found to vary from 0.9%-34.9% in Jaats. In Vaishya, besides HC and WHtR, age was found to influence BMI, WC and systolic blood pressure. In the same vein, marital status on total-C; smoking on HDL-C; types of the family on triglycerides and LDL-C; education on WHR, triglycerides, glucose and LBM and diet on WHR were found to influence these variables. The qualitative categorical variables were found to contribute 0.9%-28.7% variation in the quantitative variables in Vaishya. These observations suggest that different categorical variables were found to influence the different quantitative variables in both ethnic groups. A significantly higher prevalence of T2DM was observed in Jaats against Vaishya in the present study. In CAD patients of Iran, a higher prevalence of T2DM was observed in the ethnic group of Gilak (38.4%) than Mazani (37.6%), Fars (34.5%),Tork (33.6%) and Lor (29.3%) groups [7] and another study showed a higher prevalence of T2DM in CAD patients of Malay(52.2%) compared to Indian(51.5%), Chinese(33.2%) and White(20.3%) ethnic groups [12]. Prevalence of smoking was not significantly different in the present study but was higher in Jaats when compared to Vaishya. In Singapore and Netherland study, the prevalence of smoking was higher in Asian ethnic groups (Indians: 46.4%, Malay: 39.6% and Chinese: 34.2%) than White (21.7%) [12]. In Iranian study, the prevalence of current smoking was higher in Fars (27 %) when compared to Kord (27%), Tork, Lor (25.7%), Gilak (20.1%) and Mazani (19.6%) [7]. In the present study, the prevalence of hypertension in the Jaats was higher when compared to Vaishya but was not statistically significant as found in an earlier study [12]. In Iranian study, the prevalence of hypertension was higher in Mazani (60.8%) followed by Tork (60.7%), Lor (59.5%), Gilak (58%) and Kord (55.9%) ethnic groups [7]. Dyslipidemia was highly prevalent in Vaishya than Jaats in the present study but was not statistically significant. In Singapore and Netherland study, the prevalence of dyslipidemia was higher in Indians (77.5%), Malay (75.4%) and Chinese (70.5%) against White (48.1%) [12]. In Iranian study, prevalence of hyperlipidemia was higher in Gilak (71.7%) followed by Mazani (68.9%), Fars (66.4%), Tork (66.3%), Kord (62.7%) and Lor (62.6%)[7].In the present study, equal percent of patients in both ethnic groups had positive family history of CAD. In the Iranian study, a higher percentage of positive family history of CAD was noticed in Fars (22.2%) followed by Gilak (22%), Mazani (19.7%), Kord (18.2%), Lor (16.6%) and Tork (16.3%) [7]. Prevalence of combined positive family history of hypertension, T2DM and general obesity was significantly higher in Vaishya when compared to Jaats in the present study. This observation is only reported in our study.Prevalence of general obesity, high total-C, high LDL-C and high total-C/HDL-C was found to be significantly higher in Vaishya against Jaats in contrast prevalence of hypertriglyceridemia was significantly higher in Jaats when compared to Vaishya in the present study and reported in our study only. The results of the present study suggest clustering of T2DM and hypertriglyceridemia in Jaats and general obesity, high total-C, high LDL-C and high total-C/HDL-C and combined positive family history of hypertension, general obesity and T2DM in Vaishya. Univariate logistic regression analyses were done to find out the association of respective CRFs (dependent variable)(T2DM,isolated hypertriglyceridemia, general obesity, high total-C, high LDL-C and total-C/HDL-C) and ethnic group as an independent variable. In univariate logistic regression analyses, significant association of Jaat ethnic group with T2DM (B=0.880, OR 2.425 95% CI 1.045-5.626, P=0.039) and isolated hyper triglyceridemia (B=1.239, OR 3.451 95% CI 1.220-9.759, P=0.020). Risk factors such as general obesity(B= -1.895, OR 0.150 95% CI 0.057-0.397, P=0.000), high total-C(B= -1.287, OR 0.276 95% CI 0.119-0.642, P=0.003), high LDL-C(B=-1.088, OR 0.337 95% CI 0.148-0.767, P=0.010); and total-C/HDL-C (B= -0.824, OR 0.439 95% CI 0.196-0.984, P=0.046) were significantly associated with Vaishya ethnic group.

## Conclusion:

Significant differences in the distribution of coronary risk factors as well as the components of risk factors (mean values) were observed between CAD patients of ethnic groups in the present study. Within each ethnic group clustering of coronary risk factors was found to be different and also the effect of qualitative categorical variables on the quantitative variables. Population-specificapproach of identifying high-risk groups and designing of interventions may yield good results in reducing the complications and management of CAD. Case-control studies in ethnic groups may enlighten us on the strength of association between coronary risk factors and CAD which are in progress.

## Figures and Tables

**Table 1:** Descriptive statistics of investigated CAD patients of two caste populations

Variables	Jaats (n=50)	Vaishya(n=50)	P value
Age (years)	51.40 ± 12.93	49.74 ± 12.23	0.51
Height (cm)	173.90 ± 7.01	168.42 ± 5.17	0
Weight (Kg)	74.92 ± 6.72	75.24 ± 5.52	0.79
BMI (Kg/m2)	24.80 ± 2.09	26.54 ± 1.89	0
Waist circumference (cm)	104.38 ±12.56	95.88 ± 6.42	0
Hip circumference (cm)	112.90 ± 11.77	104.60 ± 6.40	0
Waist-hip ratio	0.92 ± 0.02	0.91 ± 0.01	0.08
Total cholesterol (mg/dl)	177.58 ± 51.65	207.28 ± 53.97	0
Triglycerides (mg/dl)	186.82 ± 120.25	182.26 ± 82.56	0.82
High Density Lipoprotein cholesterol (mg/dl)	38.12 ± 10.30	40.58 ± 8.02	0.18
Low Density Lipoprotein cholesterol (mg/dl)	117.62 ± 44.05	133.95 ± 40.59	0.057
Total-C/HDL-C ratio	4.72 ±1.66	5.17 ± 1.40	0.14
Glucose (mg/dl)	218.14 ± 35.75	197.72 ± 38.71	0
Systolic blood pressure (mmHg)	153.50 ± 26.15	153.50 ± 26.15	1
Diastolic blood pressure (mmHg)	96.20 ± 13.79	96.20 ± 13.79	1
LDL-C/HDL-C ratio	3.21 ± 1.23	3.40 ± 1.14	0.42
Waist-height ratio	0.60 ± 0.07	0.56 ± 0.04	0.01

**Table 2:** Categorical variables of CAD patients of two endogamous populations

Variables	Jaat (n=50)	Vaishya(n=50)
Age groups(years)		
21-40	8(16.00)	15(30.00)
>40	42(84.00)	35(70.00)
Smoking	37(74.00)	34(68.00)
Alcoholism	37(74.00)	38(76.00)
Marital status		
Unmarried	2(4.00)	2(4.00)
Married	39(78.00)	44(88.00)
Widower	9(18.00)	4(8.00)
Types of family		
Nuclear	7(14.00)	23(46.00)
Joint	43(86.00)	26(52.00)
Extended	0(0.00)	1(2.00)
Occupation		
Agriculture	7(14.00)	0(0.00)
Government	14(28.00)	14(28.00)
Private	12(24.00)	19(38.00)
Business	15(30.00)	17(34.00)
Unemployed	2(4.00)	0(0.00)
Education		
Middle school	1(2.00)	0(0.00)
High school	6(12.00)	2(4.00)
Intermediate/diploma	10(20.00)	5(10.00)
Graduate	25(50.00)	32(64.00)
Professional or Honours		
	8(16.00)	11(22.00)
Family monthly Income (Rs)		
30,831-46,128		
46129-61,662	7(14.00)	3(6.00)
61,663-123,321	33(66.00)	41(82.00)
	10(20.00)	6(12.00)
Socio-economic class		
Upper	3(6.00)	3(6.00)
Upper middle	47(94.00)	47(94.00)
Physical activity		
No exercise	3(6.00)	2(4.00)
Occasional exercise	35(70.00)	26(52.00)
Regular exercise	12(24.00)	22(44.00)
Tobacco chewing		
No	29(58.00)	32(64.00)
Gutka	13(26.00)	13(26.00)
Pan masala	4(8.00)	5(10.00)
Betel chewing	4(8.00)	0(0.00)
Diet		
Vegetarian	14(28.00)	20(40.00)
Non-vegetarian	36(72.00)	30(60.00)
WHR based obesity	47(94.00)	49(98.00)
Hypertension	41(82.00)	39(78.00)
High total cholesterol	13(26.00)	28(56.00)
High triglycerides	23(46.00)	30(60.00)
Low HDL-C	29(58.00)	23(46.00)
High LDL-C	15(30.00)	28(56.00)
High total-C/HDL-C	23(46.00)	33(66.00)
Type 2 diabetes	37(74.00)	27(54.00)
Positive family history of CAD	25(50.00)	25(50.00)
Combined positive	11(22.00)	20(40.00)
amily history of hypertension,		
type 2 diabetes and		
general obesity		
Waist based obesity	44(88.00)	40(80.00)
High LDL-C/HDL-C ratio	17(34.00)	23(46.00)
Isolated hypercholesterolemia	4(8.00)	3(6.00)
Isolated hypertriglyceridemia	16(32.00)	5(10.00)
Isolated low HDL-C	10(20.00)	7(14.00)
Higher non-HDL-C	50(100.00)	50(100.00)
Dyslipidemia	40(80.00)	44(88.00)
General obesity	24(48.00)	43(86.00)
C-Cholesterol
